# An important role of podoplanin in hair follicle growth

**DOI:** 10.1371/journal.pone.0219938

**Published:** 2019-07-23

**Authors:** Sun-Young Yoon, Lothar C. Dieterich, Carlotta Tacconi, Marko Sesartic, Yuliang He, Lorenz Brunner, Ohsang Kwon, Michael Detmar

**Affiliations:** 1 Institute of Pharmaceutical Sciences, Swiss Federal Institute of Technology, ETH Zurich, Zurich, Switzerland; 2 School of Pharmacy, Sungkyunkwan University, Suwon, Republic of Korea; 3 Department of Dermatology, Seoul National University College of Medicine, Seoul, Republic of Korea; Yale University School of Medicine, UNITED STATES

## Abstract

Podoplanin (PDPN) is a glycoprotein that is expressed by various cell types, including keratinocytes, fibroblasts, and lymphatic endothelial cells. We found that PDPN is expressed in the hair follicle (HF) keratinocyte region and HF stem cell area during the late anagen phase but not during the telogen phase in mice. Importantly, keratinocyte-specific PDPN deletion in mice (K5-Cre;PDPN^flox/flox^) promoted anagen HF growth after depilation-induced HF regeneration as compared to control mice. RNA sequencing, followed by gene ontology analysis, showed down-regulation of focal adhesion and extracellular matrix interaction pathways in HF stem cells isolated from K5-Cre;PDPN^flox/flox^ mice as compared to control mice. Furthermore, HF keratinocytes isolated from K5-Cre;PDPN^flox/flox^ mice exhibited a decreased ability to interact with collagen type I in cell adhesion assays. Taken together, these results show that PDPN deletion promotes HF cycling, possibly via reduced focal adhesion and concomitantly enhanced migration of HF stem cells towards the bulb region. They also indicate potential new therapeutic strategies for the treatment of conditions associated with hair loss.

## Introduction

Podoplanin (PDPN) is a 38-kDa mucin-type transmembrane glycoprotein consisting of a heavily glycosylated extracellular domain, a single transmembrane domain, and a short, nine amino acid cytoplasmic tail [1, 2]. Expression of PDPN is upregulated in various tumor types, including squamous cell carcinoma, angiosarcoma, hemangioblastoma, malignant mesothelioma, and brain tumors [3–5], and correlates with increased tumor cell motility and metastasis [6]. While PDPN expression on cancer-associated fibroblasts (CAFs) of human lung adenocarcinomas is associated with poor prognosis [7–9], increased PDPN expression in human colorectal CAFs was a significant indicator of good prognosis [10]. Although not detected in the normal interfollicular epidermis, PDPN is expressed in epidermal keratinocytes during wound healing, in psoriasis and during mouse skin carcinogenesis [11–13]. PDPN is also expressed in the basal cell layer of the sebaceous gland and the outer root sheath cells of hair follicle (HF) keratinocytes [14]. The HF is a mini-organ of the skin that continuously cycles through rapid growth (anagen phase), apoptosis-driven regression (catagen phase) and relative quiescence (telogen phase) [15]. HF stem cells, which reside within the bulge region of the HF, promote the repetitive regeneration of the follicle during the hair cycle [16]. The role of PDPN in the regulation of HF cycling has remained unknown. In this study, we first characterized PDPN expression throughout the depilation-induced hair cycle in mice. We next investigated whether keratinocyte-specific PDPN deletion in mice (K5-Cre;PDPN^flox/flox^ mice) might have an effect on HF growth. To identify the molecular and cellular mechanisms, we also performed RNA sequencing analysis of HF stem cells isolated from K5-Cre;PDPN^flox/flox^ and control mice. Our results reveal that PDPN regulates HF cyling.

## Materials and methods

### Ethics statement

This study was approved by the Institutional Review Board at the Seoul National University Hospital (approval number C-1203-031-400), and all subjects provided written informed consents. All experimental procedures using human materials were conducted according to the principles described in the Declaration of Helsinki.

### Mouse models

To generate K5-Cre;PDPN^flox/flox^ knockout mice on the C57BL/6 background, we crossed keratin 5 (K5)-Cre-ERT2 mice obtained from the MMRRC repository (University of Missouri, USA) [17] with PDPN floxed (PDPN^flox/flox^) mice (Ozgene Pty Ltd, Perth Australia). PDPN^flox/flox^ mice were designed with the loxP sites flanking exon 2 and were used as control mice. To investigate the effects of PDPN on HF keratinocytes during the hair cycle after depilation-induced hair regeneration, the back skin of 8-week-old female K5-Cre;PDPN^flox/flox^ mice in the telogen phase was depilated using wax as described [15, 18], resulting in the synchronized induction of new anagen follicle growth. The number of mice used for the experiment is indicated in the figure legends. Mice were sacrificed with an overdose of anaesthesia (160 mg kg^−1^ ketamine; 0.4 mg kg^−1^ medetomidine) at days 2, 5, 8, 19, and 22 after depilation, and back skin was taken for histological analysis. To measure the bulb diameter, 3 images/mouse were acquired of hematoxylin and eosin (H&E)-stained paraffin sections and the bulb diameter was measured at the level of the largest diameter (“Auber’s line”) of the hair bulbs with a clearly visible dermal papilla (DP) [19]. For quantitative analysis, the Image J software (National Institutes of Health, Bethesda, Maryland, USA) was used. All experimental procedures were conducted according to animal protocols approved by the Kantonales Veterinaeramt Zuerich.

### Immunofluorescence staining

Back skin samples were embedded in OCT compound (Sakura Finetek, Tokyo, Japan) and frozen in liquid nitrogen. 10-μm frozen sections were fixed in 4% paraformaldehyde for 15 min at room temperature (RT), washed in phosphate-buffered saline (PBS), and incubated with blocking solution (5% donkey serum, 0.2% bovine serum albumin, and 0.3% Triton X-100 in PBS) for 1 h at RT. Next, the sections were stained with primary antibodies overnight at 4°C and, after several washes, incubated with secondary antibodies for 30 min at RT. Primary antibodies were as follows: rabbit anti-LYVE-1 (Angiobio, Del Mar, CA, USA), goat anti-podoplanin (R&D Systems, Minneapolis, MN, USA), rabbit anti-cytokeratin 15 (Abcam, Cambridge, MA, USA), mouse anti-COL1A1 (Novus biologicals, Colorado, USA), rat anti-TNXB (Novus biologicals), mouse anti-ITGB3 (Santa Cruz Biotechnology, Dallas, TX, USA) and rat anti-CD34 (BD Biosciences, San Jose, CA, USA). Secondary antibodies (all from ThermoFisher, San Jose, CA, USA) were as follows: donkey anti-rabbit Alexa Fluor 488, donkey anti-rat Alexa Fluor 488, donkey anti-goat Alexa Fluor 594, goat anti-rat Alexa Fluor 594, goat anti-mouse Alexa Fluor 594, goat anti-rabbit Alexa Fluor 488. Hoechst 33342 (Invitrogen, Carlsbad, CA, USA) was used for nuclear staining. Immunofluorescence images were acquired by an Axioskop 2 mot plus microscope (Carl Zeiss, Jena, Germany) and high magnification images were obtained using a Zeiss LSM 710 FCS confocal microscope.

### Isolation of HF stem cells from mouse back skin

At day 12 (late-anagen phase) after depilation, the back skin of control and K5-Cre;PDPN^flox/flox^ mice (n = 5 each) was dissected, minced using scissors, and digested in Dulbecco's Modified Eagle Medium supplemented with 2% fetal bovine serum (FBS), 1.2 mM CaCl_2_, 3.5 mg/ml collagenase IV (Gibco, Grand Island, NY, USA), and 40 μg/ml DNase I for 20 min at 37°C under constant rotation. Samples were passed through a 70-μm cell strainer and washed with FACS buffer (PBS, 1% FBS, 2 mM ethylenediamine tetraacetic acid). After spinning down the cells (10 min, 1200 rpm), cell pellets were resuspended with FACS buffer and passed through a 40 μm cell strainer. Cells were stained with antibodies for 20 min. Antibodies were as follows: CD49f-APC (Integrin α6, eBioscience, San Diego, CA, USA), CD34-FITC (BD Pharmingen, San Jose, CA, USA), CD31-PE (Biolegend, San Diego, CA, USA), and CD45-APC/Cy7 (Biolegend). For live/ dead discrimination, 7-AAD (Biolegend) was used. 7AAD^-^, CD45^-^, CD31^-^, CD34^+^, integrin α6^+^ cells were considered HF stem cells and were sorted using a FACS Aria 2 instrument (BD Pharmingen).

### RNA sequencing

Total RNA was isolated from the sorted cells using the RNeasy Plus Micro Kit (Qiagen, Hilden, Germany) and RNA quality was assessed using bioanalyzer (Agilent Technologies, Santa Clara, CA, USA). For the preparation of sequencing libraries, RNA was reverse transcribed to double-stranded cDNA and then amplified using the NuGEN Ovation RNA-Seq System according to the manufacturer’s instructions. The quality of libraries was assessed with the High Sensitivity D1000 ScreenTape system (Agilent). For sequencing, Illumina HiSeq 2500 v4 was used to generate paired-end reads of 126 nt length. For data processing, the raw reads were first cleaned by removing adapter sequences, trimming low quality ends, and filtering reads with low quality (phred quality <20) using Trimmomatic [20]. Sequence alignment of the resulting high-quality reads to the *Mus musculus* reference genome (build GRCm38) was carried out using STAR (Version 2.5.1b) [21]. Gene expression values were computed with the function featureCounts from the Bioconductor package Rsubread [22]. Differential expression analysis was performed using the generalized linear model implemented in the Bioconductor package EdgeR [23]. Differential expression was assessed using an exact test adapted for over-dispersed data. Genes showing altered expression with adjusted (Benjamini and Hochberg method) p-value < 0.05 were considered differentially expressed.

### Isolation of HF keratinocytes from mouse back skin and cell culture

The methods used for isolation of mouse HF keratinocytes have been described previously [24]. At postnatal day 2, back skin samples were obtained from K5-Cre;PDPN^flox/flox^ and control mice and digested in 0.8% trypsin solution (Sigma-Aldrich, St. Louis, MO, USA) with shaking at 75 rpm for 1 h at 37°C. The epidermis was peeled away, minced using scissors, and incubated in Dulbecco's Modified Eagle Medium supplemented with 30 μg/ml DNase solution (AppliChem, Darmstadt, Germany) and antibiotic/antimycotic solution (Gibco) for 30 min at 37°C under constant rotation. Samples were passed through a 100-μm cell strainer and washed with PBS including 1% FBS and 2 mM ethylenediamine tetraacetic acid. After spinning down the cells (7 min, 1200 rpm), pellets were resuspended with culture medium and cultured on 25 μg/ml type IV collagen (collagen from human placenta, Sigma-Aldrich)-coated 6-well plates. Culture medium consisted of dF-medium (defined keratinocyte-SFM (Invitrogen), 1 ml growth supplement (Invitrogen), 1% penicillin/streptomycin (Invitrogen), and 10^−10^ M cholera toxin (Sigma-Aldrich)) and KGF-medium (Minimum Essential Medium Eagle (MEM, Sigma-Aldrich), 5 μg/ml insulin (Sigma-Aldrich), 10 μg/ml transferrin (Sigma-Aldrich), 1.4 μg/ml phosphoethanolamine (Sigma-Aldrich), 10 mM ethanolamine (Sigma-Aldrich), 0.36 μg/ml hydrocortisone (Calbiochem, La Jolla, CA), 1% glutamine (Invitrogen), 1% penicillin/streptomycin (Invitrogen), 8% chelated FCS (Bio-Rad, Hercules, CA, USA), and 6.6 μg/ml CaCl_2_ (Merck, Darmstadt, Germany)) at a ratio of 2:1 supplemented with 10 ng/ml epidermal growth factor (Sigma-Aldrich). At day 5 of culture, we confirmed that living cells consisted of more than 90% HF keratinocytes (Zombie-NIR^-^, CD45-Percp^-^, CD90-FITC^-^ and integrin α6-APC^+^ cells) using FACS (CytoFLEX, Beckman Coulter, Pasadena, CA, USA).

### Cell adhesion assays

HF keratinocytes isolated from K5-Cre;PDPN^flox/flox^ and control mice were labelled with 6 μM calcein for 10 min at 37°C and then washed twice in PBS. Calcein-labelled keratinocytes (10^4^ cells/well) were seeded on 25 μg/ml type I collagen (PureCol, Advanced BioMatrix, San Diego, CA, USA) or 0.1% bovine serum albumin (negative control)-coated 96-well plates (black, clear bottom, Corning, NY, USA) and incubated for 1 h at 37°C. After washing twice with PBS, 100 μl PBS was added to each sample and the measurement of fluorescence was performed using a Spectramax reader (excitation: 485 nm and emission: 538 nm, Molecular Devices).

### Scratch wound healing assays

The methods used for a scratch wound healing closure assay have been described previously [25, 26]. Mouse HF keratinocytes isolated from K5-Cre;PDPN^flox/flox^ and control mice (at postnatal day 4) were grown to full confluence in 25 μg/ml type IV collagen (collagen from human placenta, Sigma-Aldrich)-coated 24-well plates and were then incubated overnight in serum-reduced medium containing 1% FBS. Cells were scratched with a 200 μL sterile pipette tip and were then incubated. After 48 h, cells were washed with PBS and the images of the scratches were acquired. The surface areas of the cell-free zones were measured and the % scratch closure was determined using TScratch software [26].

### Quantitative real time-polymerase chain reaction (qRT-PCR)

Total RNA was isolated using the NucleoSpin RNA kit (Macherey-Nagel, Düren, Germany) and 1 μg of total RNA was used for the cDNA synthesis using the High Capacity Reverse Transcription kit (Applied Biosystems, Foster City, CA, USA). PCR was performed on a 7900HT Fast Real-Time PCR System (Applied Biosystems) using FastStart SYBR green master mix (Roche Diagnostics, Basel, Switzerland) according to the manufacturer’s instructions. Gene expression was normalized to the control gene GAPDH. Primer information is provided in S1 Table.

### Statistical analyses

Statistical significance was determined using a two-tailed unpaired t-test (GraphPad Prism, version 5.0a, San Diego, CA, USA) and results were considered significant at a p-value < 0.05.

## Results

### PDPN is expressed in the HF keratinocyte region and HF stem cell area during the late anagen but not the telogen phase

**Fig 1:**
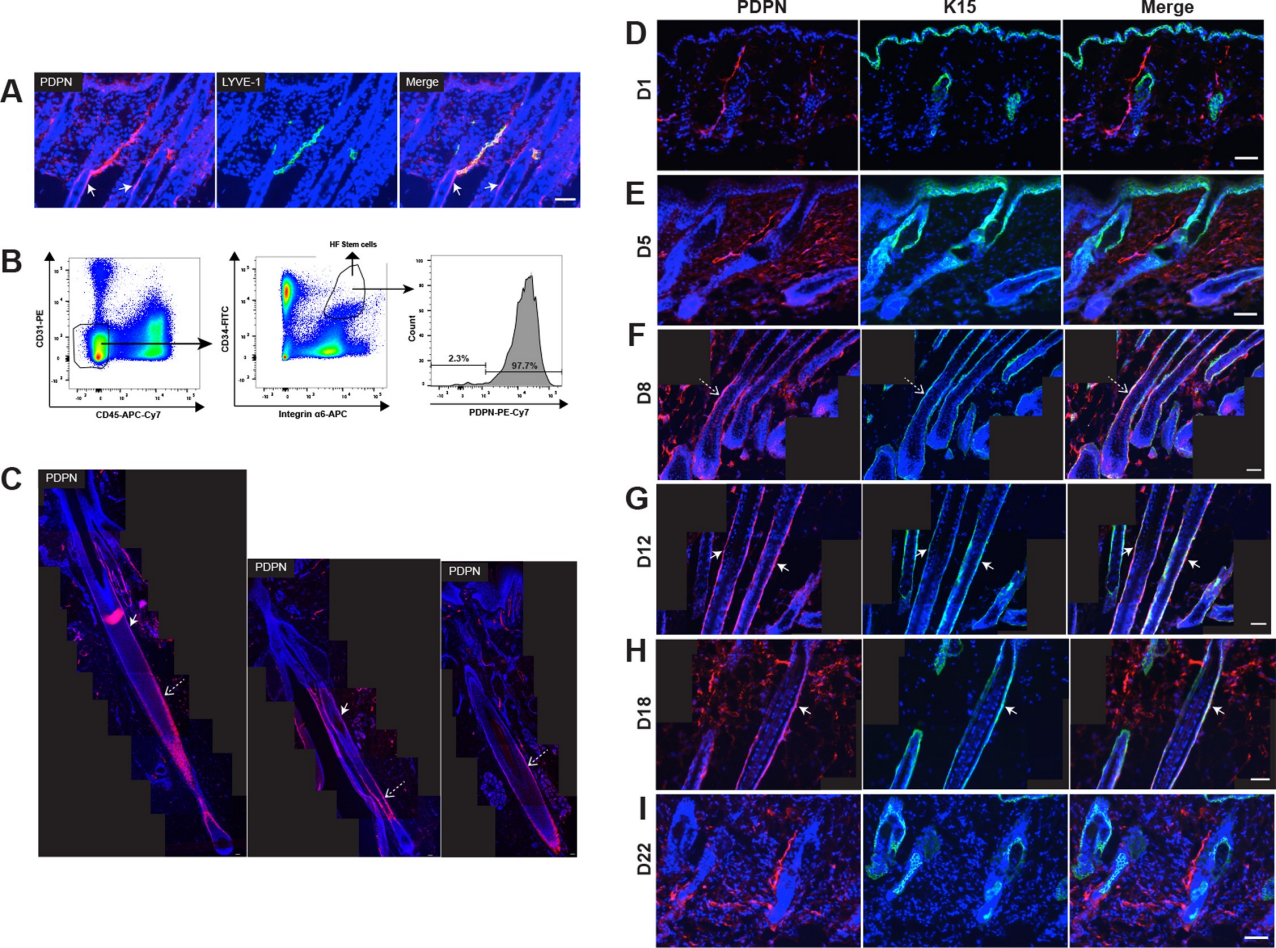
PDPN is expressed in the HF keratinocyte region and the stem cell area during the late anagen but not the telogen phase. (**A**) Immunofluorescence staining of 10-μm frozen sections of back skin (anagen growth phase, C57BL/6J female mice at day 12 after depilation) for PDPN (red) and LYVE-1 (lymphatic marker, green). Nuclear staining with Hoechst 33342 (blue). (**B**) FACS gating strategy for sorting HF stem cells. Left panel: Skin-derived cell suspension pre-gated for living (7AAD^-^) singlets. Middle panel: CD45^-^ CD31^-^ CD34^+^ integrin α6^+^ cells were considered as HF-stem cells. Right panel:PDPN^+^ cells were dectected in HF stem cells. (**C**) Immunofluorescence stainings of 10-μm paraffin sections for PDPN (red) in human scalp tissue. Nuclear staining with Hoechst 33342 (blue). (**D-I**) After depilation-induced HF regeneration in C57BL/6J female mice (n = 4 each group), back skin samples were obtained at days 1 (early-anagen phase), 5 (mid-anagen phase), 8 (late-anagen phase), 12 (late-anagen phase), 18 (catagen phase), and 22 (telogen phase). Double immunofluorescence stainings of 10-μm frozen sections of back skin for PDPN (red) and K15 (green). Nuclear staining with Hoechst 33342 (blue). (**C, F, G, and H**) Tiled images from each immunofluorescence image were created to visualize large fields. White arrows indicate the bulge area. Dashed arrows indicate the HF keratinocyte region. Scale bars: 50 μm.

To characterize the location of PDPN in back skin samples of wild-type mice in the anagen phase, we performed double immunofluorescence stainings for the lymphatic vessel (LV) marker LYVE-1 and for PDPN. PDPN was co-expressed by LYVE-1-positive LVs and was especially located in anagen HFs, in particular in outer root sheath cells and the HF stem cell area (Fig 1A). To further characterize PDPN in the HF, back skin samples were stained for keratin 15 and CD34, which are putative markers of HF stem cells. To isolate HF stem cells from mouse back skin using fluorescence-activated cell sorting (FACS), antibodies for CD34 and integrin α6, putative markers of HF stem cells, were used. PDPN-positive cells were observed in HF stem cells (CD45-, CD31-, CD34+, integrin α6+ cells) (Fig 1B). PDPN was also detected in LVs, HF keratinocytes and HF stem cells of human scalp tissue (Fig 1C). These results indicate that PDPN is expressed in HFs, particularly in the HF keratinocyte region and HF stem cell area.

We next investigated by double immunofluorescence staining for PDPN and K15 whether PDPN expression might undergo cyclic changes during depilation-induced hair regeneration in female C57BL/6J mice. At day 1 (early-anagen phase) and day 5 (mid-anagen phase) after depilation, PDPN was expressed in LVs but was absent from HF keratinocytes (Fig 1D and 1E). Interestingly, at days 8 and 12 (late-anagen phase), PDPN was expressed in HF keratinocytes and HF stem cells (Fig 1F and 1G). At day 18 (catagen phase), PDPN expression was still present in HF keratinocytes (Fig 1H and 1D). However, at day 22 (telogen phase), PDPN was detected in LVs but not HFs (Fig 1I). These results indicate that PDPN is expressed in HF keratinocytes and HF stem cells during the late-anagen growth phase but not during the telogen phase, suggesting that PDPN might be involved in HF cycling.

### K5-Cre;PDPN^flox/flox^ mice show enhanced anagen growth

**Fig 2:**
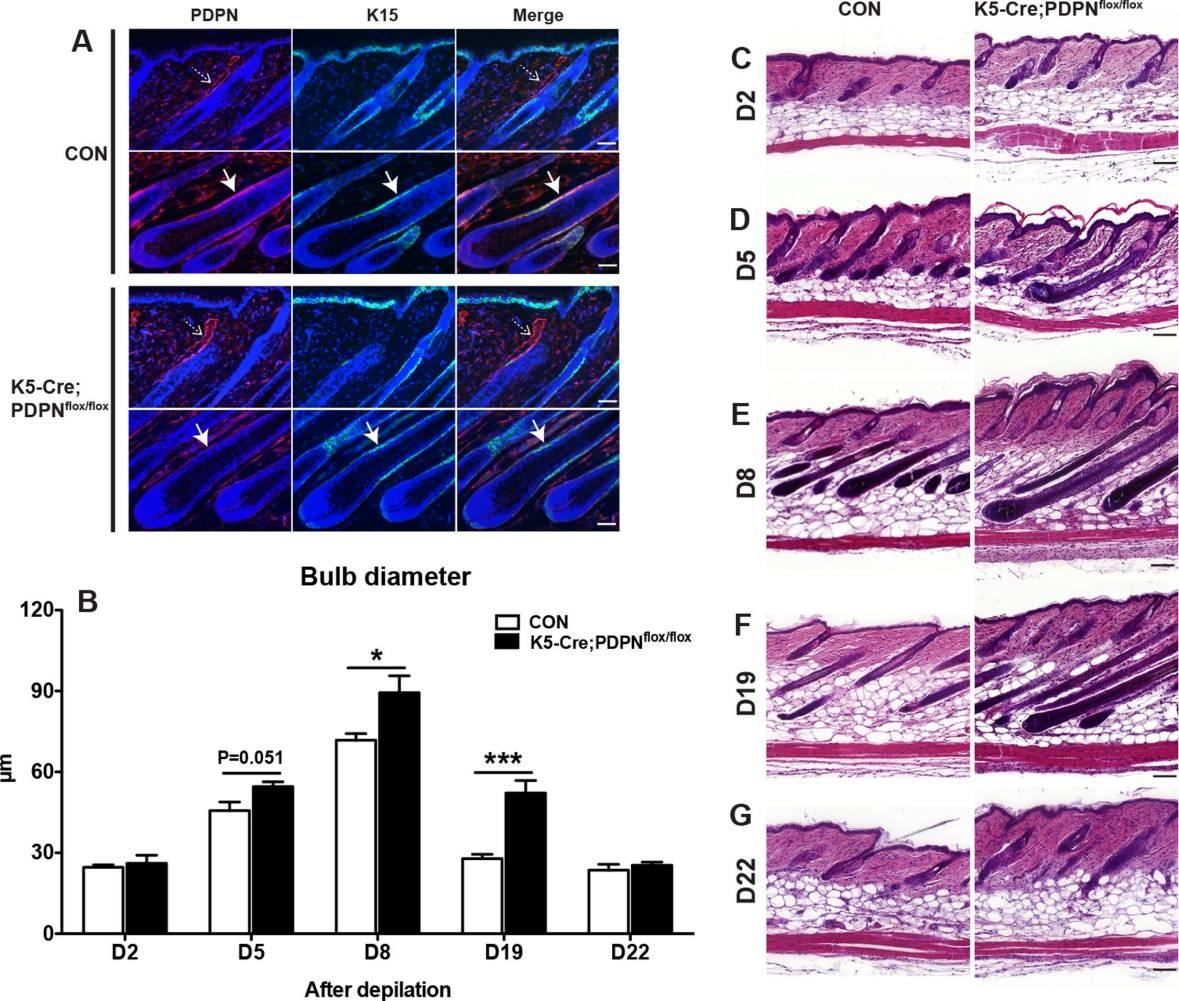
K5-Cre;PDPN^flox/flox^ mice have increased anagen HF growth. (**A**) Double immunofluorescence stainings of 10-μm back skin frozen sections for PDPN (red) and K15 (green) in control and K5-Cre;PDPN^flox/flox^ mice (day 12 after depilation). Nuclear staining with Hoechst 33342 (blue). White arrows indicate the HF keratinocyte region. Dashed arrows indicate the lymphatic vessels. (**B**) In H&E stained paraffin sections, 3 images/mouse were acquired and the bulb diameter was measured at the level of the largest diameter (“Auber’s line”). Data were analyzed using the two-tailed unpaired t-test for each time point. Results are presented as mean ± standard error of mean (SEM). ***P < 0.001, *P < 0.05 versus control group. (**C**-**G**) After depilation-induced HF regeneration, back skin samples were obtained at days 2 (**C**, CON: n = 4, K5-Cre;PDPN^flox/flox^: n = 5), 5 (**D**, CON: n = 4, K5-Cre;PDPN^flox/flox^: n = 4), 8 (**E**, CON: n = 5, K5-Cre;PDPN^flox/flox^: n = 4), 19 (**F**, CON: n = 8, K5-Cre;PDPN^flox/flox^: n = 10), and 22 (**G**, CON: n = 3, K5-Cre;PDPN^flox/flox^: n = 4), and paraffin sections were stained with H&E. Data were analyzed using the two-tailed unpaired t-test. Scale bars: 50 μm (**A**); 100 μm (**C-G**).

Given the cyclic changes of PDPN expression during the hair cycle, we next investigated whether keratinocyte-specific PDPN deletion in mice might have an effect on HF growth. We first studied depilation-induced hair regeneration in keratinocyte-specific PDPN deleted mice (K5-Cre;PDPN^flox/flox^). Double immunofluorescence stainings for PDPN and K15 confirmed the absence of PDPN expression in HF keratinocytes of K5-Cre;PDPN^flox/flox^ mice, whereas PDPN was located in HF keratinocytes of littermate PDPN^flox/flox^ control mice (Fig 2A). Since the thickness of the hair shaft is known to correlate with the size of the hair bulb [27], we measured the diameter of the hair bulbs during depilation-induced hair regeneration, which typically increases during the anagen phase, whereas it is reduced during catagen development [15]. At day 2 (early-anagen phase) after depilation, the diameter of the hair bulb was comparable in both control and K5-Cre;PDPN^flox/flox^ mice (Fig 2B and 2C), but started to become larger in K5-Cre;PDPN^flox/flox^ mice than in control mice at day 5 (mid-anagen phase) (Fig 2B and 2D). Importantly, at days 8 (mid-anagen phase) and 19 (catagen phase), the thickness of hair bulbs was significantly larger in K5-Cre;PDPN^flox/flox^ mice than in control mice (Fig 2B, 2E and 2F). At day 22 (telogen phase), the hair bulb diameter was comparable in both control and K5-Cre;PDPN^flox/flox^ mice. Together, these results indicate that after depilation, K5-Cre;PDPN^flox/flox^ mice have increased anagen HF growth compared to control mice.

### Down-regulated focal adhesion in HF stem cells isolated from K5-Cre;PDPN^flox/flox^ mice

**Fig 3:**
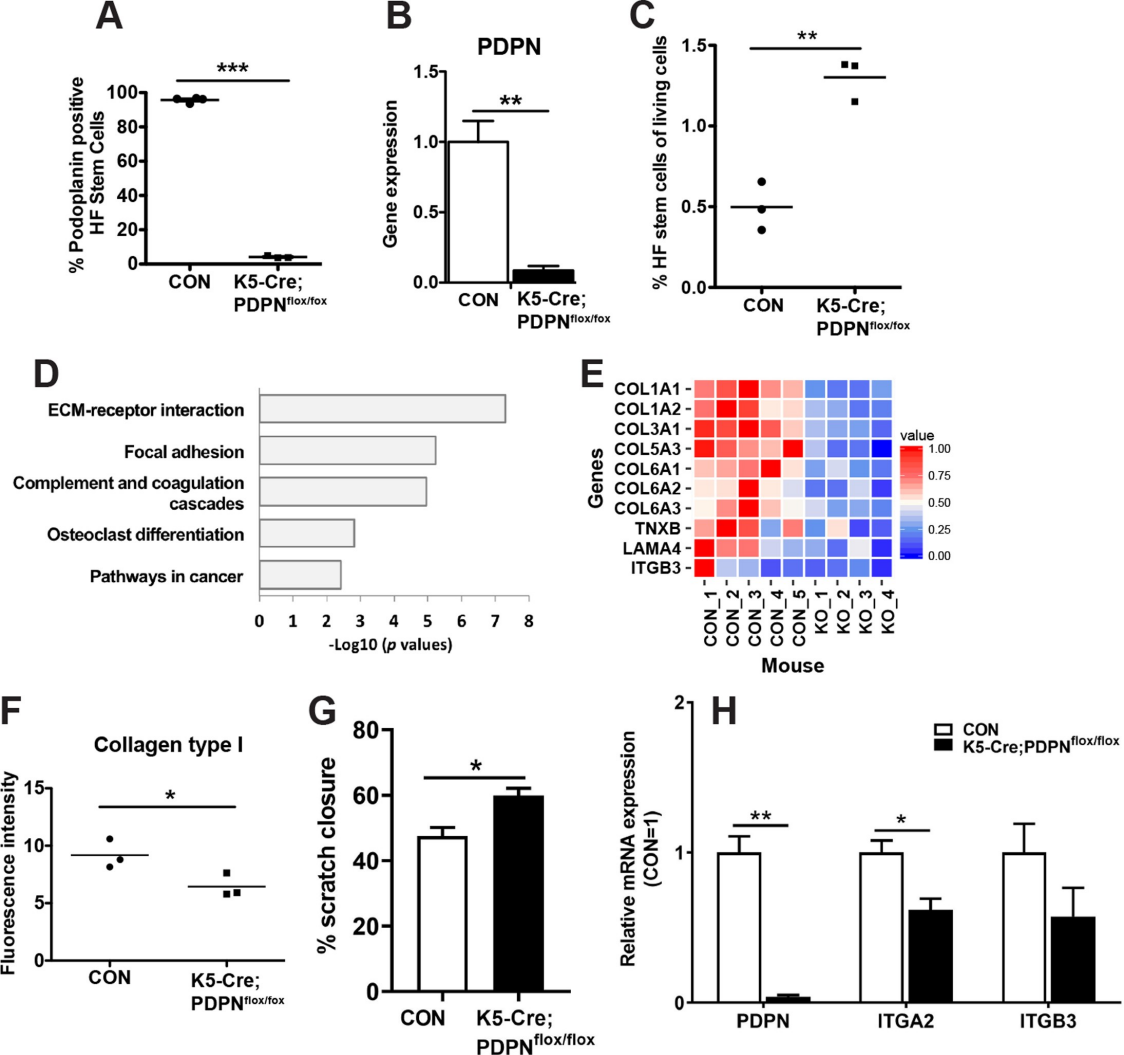
Reduced matrix adhesion of HF stem cells isolated from K5-Cre;PDPN^flox/flox^ mice. (**A**) At day 12 (late-anagen growth phase) after depilation (CON: n = 4, K5-Cre;PDPN^flox/flox^: n = 3), the percentage of PDPN^+^ cells among the HF stem cells was determined by FACS. **(B**) Expression of PDPN in HF stem cells isolated from CON and K5-Cre;PDPN^flox/flox^ mice was determined by RNA sequencing (CON: n = 5, K5-Cre;PDPN^flox/flox^: n = 4). Results are presented as mean ± SEM. (**C**) At day 12 (late-anagen growth phase) after depilation (CON; n = 3, K5-Cre;PDPN^flox/flox^: n = 3), the percentage of HF stem cells among the living cells was determined by FACS. (**D**) Gene ontology analysis of down-regulated genes in HF stem cells isolated from K5-Cre;PDPN^flox/flox^ mice. (**E**) Heat map indicating down-regulated genes based on the focal adhesion and extracellular matrix receptor interaction pathways in these cells from K5-Cre;PDPN^flox/flox^ mice (KO). (**F**) In HF keratinocytes isolated from CON and K5-Cre;PDPN^flox/flox^ mice, adhesion to collagen type I was examined *in vitro*. (**G**) Cell migration was assessed using a scratch wound healing closure assay in HF keratinocytes isolated from CON and K5-Cre;PDPN^flox/flox^ mice. (**H**) Total RNA was isolated from HF keratinocytes isolated from CON and K5-Cre;PDPN^flox/flox^ mice and qRT-PCR was performed. Results are presented as mean ± SEM. (**A**, **B, C**, **F, G** and **H**) Data were analyzed using the two-tailed unpaired t-test. ***P < 0.001, **P < 0.01, *P < 0.05 versus control group.

To identify potential molecular and cellular mechanisms by which PDPN deletion might promote anagen HF growth, we performed RNA sequencing of HF stem cells isolated from control and K5-Cre;PDPN^flox/flox^ mice at day 12 (late-anagen growth phase) after depilation using FACS. The RNA sequencing data were uploaded in the European Nucleotide Archive (ENA) under accession number PRJEB22837 (For reviewers access: https://www.ebi.ac.uk/ena/submit/sra/#home username “Webin-47976” and password “781228.kim”). As expected, PDPN expression was markedly decreased in HF stem cells isolated from K5-Cre;PDPN^flox/flox^ mice as compared to those from control mice using FACS (Fig 3A) and RNA sequencing (Fig 3B). Moreover, at day 12 after depilation, there were more HF stem cells in K5-Cre;PDPN^flox/flox^ mice compared to control mice (Fig 3C). Gene ontology analysis showed down-regulation of focal adhesion and extracellular matrix interaction pathways in HF stem cells isolated from K5-Cre;PDPN^flox/flox^ mice (Fig 3D). As cellular migration is associated with increased cell adhesion in epithelial cells [28, 29], these data suggest a potentially increased capacity of HF stem cells to migrate towards the bulb area. Down-regulated genes included several collagens, TNXB, LAMA4, and ITGB3 (Fig 3E). To confirm our RNA sequencing data, immunofluorescence stainings for tenascin XB (TNXB), collagen type I alpha 1 chain (COL1A1), and integrin beta 3 (ITGB3) were performed. We found that the expression of TNXB, COL1A1 and ITGB3 were weaker in K5-Cre;PDPN^flox/flox^ mice than in control mice (S1 Fig). Furthermore, HF keratinocytes isolated from K5-Cre;PDPN^flox/flox^ mice exhibited a decreased ability to interact with collagen type I in cell adhesion assays (Fig 3F). In addition, we also performed a scratch wound healing closure assay to assess cell migration. We found that cell migration was accelerated in HF keratinocytes isolated from keratinocyte-specific podoplanin deletion mice as compared to those from control mice (Fig 3G). Quantitative real-time (qRT)-PCR analysis confirmed that PDPN expression was strongly decreased in HF keratinocytes isolated from K5-Cre;PDPN^flox/flox^ mice (Fig 3H). Importantly, the mRNA expression of integrin α2 (ITGA2), a major cellular receptor for collagen type I, was significantly reduced in HF keratinocytes isolated from K5-Cre;PDPN^flox/flox^ mice (Fig 3H). These results indicate that PDPN deletion might promote hair growth, possibly via reduced focal adhesion and concomitantly enhanced migration of HF stem cells towards the bulb region.

## Discussion

While PDPN has a variety of functions including regulation of organ development, cell mobility, tumorigenesis, and metastasis, its physiological cell type-specific functions are still incompletely understood. In this study, we identified PDPN as a new player in the regulation of HF cycling.

Previously, it has been found that PDPN is associated with the down-regulation of the cell-cell adhesion protein E-cadherin in oral squamous cell carcinomas [2] and that it induces an epithelial-mesenchymal transition in Madin-Darby canine kidney type-II epithelial cells and immortalized HaCaT keratinocytes through the interaction with ERM proteins and up-regulation of RhoA activity [6]. PDPN knockdown resulted in decreased migration of human lung microvascular lymphatic endothelial cells and contributed to low RhoA-GTP levels in the scratch wound assay [30]. Furthermore, bone-specific PDPN deletion in osteocytes in mice resulted in the disruption of the osteocyte dendritic network [31]. This, PDPN has a variety of functions depending on the cell type in which it is expressed. In the present study, we found that PDPN is expressed in the HF keratinocyte region and HF stem cell area during late-anagen growth phase but not during telogen quiescence phase, indicating that PDPN might be involved in HF cycling.

It has been reported that during the telogen to anagen transition, HF stem cells are activated by dermal papilla cells and the cutaneous microenvironment, and migrate from the bulge towards the bulb region where they differentiate into various epithelial HF cell lineages [32, 33]. Enhanced focal adhesion has been shown to associate with reduced cell migration in focal adhesion kinase-deficient mice [34, 35]. Interestingly, using RNA sequencing of HF stem cells isolated from K5-Cre;PDPN^flox/flox^ mice, we found that the focal adhesion pathway was down-regulated. In agreement with these results, HF keratinocytes isolated from K5-Cre;PDPN^flox/flox^ mice had a decreased ability to interact with collagen type I in a cell adhesion assay. Moreover, we found that cell migration was accelerated in HF keratinocytes isolated from keratinocyte-specific podoplanin knockout mice as compared to those from control mice. Our results indicate that PDPN deletion in HF stem cells results in enhanced hair growth, possibly via reduced focal adhesion and concomitantly enhanced migration of hair follicle stem cells toward the bulb region.

Using RNA sequencing, we found that transcription factors such as Stat3 and PPARγ might contribute to the regulation of several down-regulated genes in HF stem cells isolated from K5-Cre;PDPN^flox/flox^ mice. It has been shown previously that keratinocyte-specific Stat3-disrupted mice show an impairment of anagen entry during the second hair cycle and subsequent hair growth [36]. Moreover, HF stem cell-specific PPARγ deletion in mice resulted in scarring alopecia [37]. Thus, further studies would be of interest to investigate the roles of Stat3 and PPARγ in K5-Cre;PDPN^flox/flox^ mice.

In conclusion, our results suggest an unanticipated role of PDPN in the HF cycle, with potential implications for therapeutic strategies to treat alopecia.

## Supporting information

S1 Fig (TIF)

S1 Table (TIF)

S1 Checklist (PDF)

## References

[1] Cueni LN, Detmar M. Galectin-8 interacts with podoplanin and modulates lymphatic endothelial cell functions. Exp Cell Res. 2009;315(10):1715–23. Epub 2009/03/10. 10.1016/j.yexcr.2009.02.021 19268462 PMC3398156

[2] Martin-Villar E, Scholl FG, Gamallo C, Yurrita MM, Munoz-Guerra M, Cruces J, et al Characterization of human PA2.26 antigen (T1alpha-2, podoplanin), a small membrane mucin induced in oral squamous cell carcinomas. Int J Cancer. 2005;113(6):899–910. Epub 2004/10/30. 10.1002/ijc.20656 . 15515019

[3] Schacht V, Dadras SS, Johnson LA, Jackson DG, Hong YK, Detmar M. Up-regulation of the lymphatic marker podoplanin, a mucin-type transmembrane glycoprotein, in human squamous cell carcinomas and germ cell tumors. Am J Pathol. 2005;166(3):913–21. Epub 2005/03/04. 10.1016/S0002-9440(10)62311-5 15743802 PMC1602360

[4] Roy S, Chu A, Trojanowski JQ, Zhang PJ. D2-40, a novel monoclonal antibody against the M2A antigen as a marker to distinguish hemangioblastomas from renal cell carcinomas. Acta Neuropathol. 2005;109(5):497–502. Epub 2005/05/03. 10.1007/s00401-005-0999-3 . 15864611

[5] Shibahara J, Kashima T, Kikuchi Y, Kunita A, Fukayama M. Podoplanin is expressed in subsets of tumors of the central nervous system. Virchows Arch. 2006;448(4):493–9. Epub 2006/01/18. 10.1007/s00428-005-0133-x . 16411134

[6] Martin-Villar E, Megias D, Castel S, Yurrita MM, Vilaro S, Quintanilla M. Podoplanin binds ERM proteins to activate RhoA and promote epithelial-mesenchymal transition. J Cell Sci. 2006;119(Pt 21):4541–53. Epub 2006/10/19. 10.1242/jcs.03218 . 17046996

[7] Kawase A, Ishii G, Nagai K, Ito T, Nagano T, Murata Y, et al Podoplanin expression by cancer associated fibroblasts predicts poor prognosis of lung adenocarcinoma. Int J Cancer. 2008;123(5):1053–9. Epub 2008/06/12. 10.1002/ijc.23611 . 18546264

[8] Kitano H, Kageyama S, Hewitt SM, Hayashi R, Doki Y, Ozaki Y, et al Podoplanin expression in cancerous stroma induces lymphangiogenesis and predicts lymphatic spread and patient survival. Arch Pathol Lab Med. 2010;134(10):1520–7. Epub 2010/10/07. 10.1043/2009-0114-OA.1 . 20923309 PMC7556323

[9] Hoshino A, Ishii G, Ito T, Aoyagi K, Ohtaki Y, Nagai K, et al Podoplanin-positive fibroblasts enhance lung adenocarcinoma tumor formation: podoplanin in fibroblast functions for tumor progression. Cancer Res. 2011;71(14):4769–79. Epub 2011/05/26. 10.1158/0008-5472.CAN-10-3228 . 21610106

[10] Yamanashi T, Nakanishi Y, Fujii G, Akishima-Fukasawa Y, Moriya Y, Kanai Y, et al Podoplanin expression identified in stromal fibroblasts as a favorable prognostic marker in patients with colorectal carcinoma. Oncology. 2009;77(1):53–62. Epub 2009/06/27. 10.1159/000226112 . 19556810

[11] Gandarillas A, Scholl FG, Benito N, Gamallo C, Quintanilla M. Induction of PA2.26, a cell-surface antigen expressed by active fibroblasts, in mouse epidermal keratinocytes during carcinogenesis. Mol Carcinog. 1997;20(1):10–8. Epub 1997/10/27. . 9328432 10.1002/(sici)1098-2744(199709)20:1<10::aid-mc3>3.0.co;2-m

[12] Honma M, Fujii M, Iinuma S, Minami-Hori M, Takahashi H, Ishida-Yamamoto A, et al Podoplanin expression is inversely correlated with granular layer/filaggrin formation in psoriatic epidermis. J Dermatol. 2013;40(4):296–7. Epub 2013/01/08. 10.1111/1346-8138.12060 . 23289735

[13] Asai J, Hirakawa S, Sakabe J, Kishida T, Wada M, Nakamura N, et al Platelets Regulate the Migration of Keratinocytes via Podoplanin/CLEC-2 Signaling during Cutaneous Wound Healing in Mice. Am J Pathol. 2016;186(1):101–8. Epub 2015/11/26. 10.1016/j.ajpath.2015.09.007 . 26597882

[14] Honma M, Minami-Hori M, Takahashi H, Iizuka H. Podoplanin expression in wound and hyperproliferative psoriatic epidermis: regulation by TGF-beta and STAT-3 activating cytokines, IFN-gamma, IL-6, and IL-22. J Dermatol Sci. 2012;65(2):134–40. 10.1016/j.jdermsci.2011.11.011 . 22189341

[15] Muller-Rover S, Handjiski B, van der Veen C, Eichmuller S, Foitzik K, McKay IA, et al A comprehensive guide for the accurate classification of murine hair follicles in distinct hair cycle stages. J Invest Dermatol. 2001;117(1):3–15. Epub 2001/07/10. 10.1046/j.0022-202x.2001.01377.x . 11442744

[16] Festa E, Fretz J, Berry R, Schmidt B, Rodeheffer M, Horowitz M, et al Adipocyte lineage cells contribute to the skin stem cell niche to drive hair cycling. Cell. 2011;146(5):761–71. 10.1016/j.cell.2011.07.019 21884937 PMC3298746

[17] Kataoka K, Kim DJ, Carbajal S, Clifford JL, DiGiovanni J. Stage-specific disruption of Stat3 demonstrates a direct requirement during both the initiation and promotion stages of mouse skin tumorigenesis. Carcinogenesis. 2008;29(6):1108–14. Epub 2008/05/06. 10.1093/carcin/bgn061 18453544 PMC2902397

[18] Paik SH, Yoon JS, Ryu HH, Lee JY, Shin CY, Min KH, et al Pretreatment of epidermal growth factor promotes primary hair recovery via the dystrophic anagen pathway after chemotherapy-induced alopecia. Exp Dermatol. 2013;22(7):496–9. Epub 2013/06/27. 10.1111/exd.12182 . 23800066

[19] Yano K, Brown LF, Detmar M. Control of hair growth and follicle size by VEGF-mediated angiogenesis. J Clin Invest. 2001;107(4):409–17. Epub 2001/02/22. 10.1172/JCI11317 11181640 PMC199257

[20] Bolger AM, Lohse M, Usadel B. Trimmomatic: a flexible trimmer for Illumina sequence data. Bioinformatics. 2014;30(15):2114–20. Epub 2014/04/04. 10.1093/bioinformatics/btu170 24695404 PMC4103590

[21] Dobin A, Davis CA, Schlesinger F, Drenkow J, Zaleski C, Jha S, et al STAR: ultrafast universal RNA-seq aligner. Bioinformatics. 2013;29(1):15–21. Epub 2012/10/30. 10.1093/bioinformatics/bts635 23104886 PMC3530905

[22] Liao Y, Smyth GK, Shi W. The Subread aligner: fast, accurate and scalable read mapping by seed-and-vote. Nucleic Acids Res. 2013;41(10):e108 Epub 2013/04/06. 10.1093/nar/gkt214 23558742 PMC3664803

[23] Robinson MD, McCarthy DJ, Smyth GK. edgeR: a Bioconductor package for differential expression analysis of digital gene expression data. Bioinformatics. 2010;26(1):139–40. Epub 2009/11/17. 10.1093/bioinformatics/btp616 19910308 PMC2796818

[24] Braun S, Hanselmann C, Gassmann MG, auf dem Keller U, Born-Berclaz C, Chan K, et al Nrf2 transcription factor, a novel target of keratinocyte growth factor action which regulates gene expression and inflammation in the healing skin wound. Mol Cell Biol. 2002;22(15):5492–505. Epub 2002/07/09. 10.1128/MCB.22.15.5492-5505.2002 12101242 PMC133949

[25] Roudnicky F, Yoon SY, Poghosyan S, Schwager S, Poyet C, Vella G, et al Alternative transcription of a shorter, non-anti-angiogenic thrombospondin-2 variant in cancer-associated blood vessels. Oncogene. 2018;37(19):2573–85. Epub 2018/02/23. 10.1038/s41388-018-0129-z 29467494 PMC5945577

[26] Geback T, Schulz MM, Koumoutsakos P, Detmar M. TScratch: a novel and simple software tool for automated analysis of monolayer wound healing assays. Biotechniques. 2009;46(4):265–74. Epub 2009/05/20. 10.2144/000113083 . 19450233

[27] Wosicka H, Cal K. Targeting to the hair follicles: current status and potential. J Dermatol Sci. 2010;57(2):83–9. Epub 2010/01/12. 10.1016/j.jdermsci.2009.12.005 . 20060268

[28] Schaeffer D, Somarelli JA, Hanna G, Palmer GM, Garcia-Blanco MA. Cellular migration and invasion uncoupled: increased migration is not an inexorable consequence of epithelial-to-mesenchymal transition. Mol Cell Biol. 2014;34(18):3486–99. Epub 2014/07/09. 10.1128/MCB.00694-14 25002532 PMC4135620

[29] Li Y, Francia G, Zhang JY. p62/IMP2 stimulates cell migration and reduces cell adhesion in breast cancer. Oncotarget. 2015;6(32):32656–68. Epub 2015/09/30. 10.18632/oncotarget.5328 26416451 PMC4741720

[30] Navarro A, Perez RE, Rezaiekhaligh MH, Mabry SM, Ekekezie, II. Polarized migration of lymphatic endothelial cells is critically dependent on podoplanin regulation of Cdc42. Am J Physiol Lung Cell Mol Physiol. 2011;300(1):L32–42. Epub 2010/11/03. 10.1152/ajplung.00171.2010 . 21036919

[31] Staines KA, Javaheri B, Hohenstein P, Fleming R, Ikpegbu E, Unger E, et al Hypomorphic conditional deletion of E11/Podoplanin reveals a role in osteocyte dendrite elongation. J Cell Physiol. 2017;232(11):3006–19. Epub 2017/05/11. 10.1002/jcp.25999 . 28488815 PMC5575468

[32] Schneider MR, Schmidt-Ullrich R, Paus R. The hair follicle as a dynamic miniorgan. Curr Biol. 2009;19(3):R132–42. 10.1016/j.cub.2008.12.005 . 19211055

[33] Mistriotis P, Andreadis ST. Hair follicle: a novel source of multipotent stem cells for tissue engineering and regenerative medicine. Tissue Eng Part B Rev. 2013;19(4):265–78. Epub 2012/11/20. 10.1089/ten.TEB.2012.0422 23157470 PMC3690091

[34] Ilic D, Furuta Y, Kanazawa S, Takeda N, Sobue K, Nakatsuji N, et al Reduced cell motility and enhanced focal adhesion contact formation in cells from FAK-deficient mice. Nature. 1995;377(6549):539–44. Epub 1995/10/12. 10.1038/377539a0 . 7566154

[35] Wozniak MA, Modzelewska K, Kwong L, Keely PJ. Focal adhesion regulation of cell behavior. Biochim Biophys Acta. 2004;1692(2–3):103–19. Epub 2004/07/13. 10.1016/j.bbamcr.2004.04.007 . 15246682

[36] Sano S, Kira M, Takagi S, Yoshikawa K, Takeda J, Itami S. Two distinct signaling pathways in hair cycle induction: Stat3-dependent and -independent pathways. Proc Natl Acad Sci U S A. 2000;97(25):13824–9. Epub 2000/11/23. 10.1073/pnas.240303097 11087819 PMC17660

[37] Karnik P, Tekeste Z, McCormick TS, Gilliam AC, Price VH, Cooper KD, et al Hair follicle stem cell-specific PPARgamma deletion causes scarring alopecia. J Invest Dermatol. 2009;129(5):1243–57. Epub 2008/12/05. 10.1038/jid.2008.369 19052558 PMC3130601

