# Supplementary figures and images for: An important role of podoplanin in hair follicle growth
Source: PLoS One. 2019 Jul 23;14(7):e0219938. doi: 10.1371/journal.pone.0219938 (PMC6650137; doi:10.1371/journal.pone.0219938)

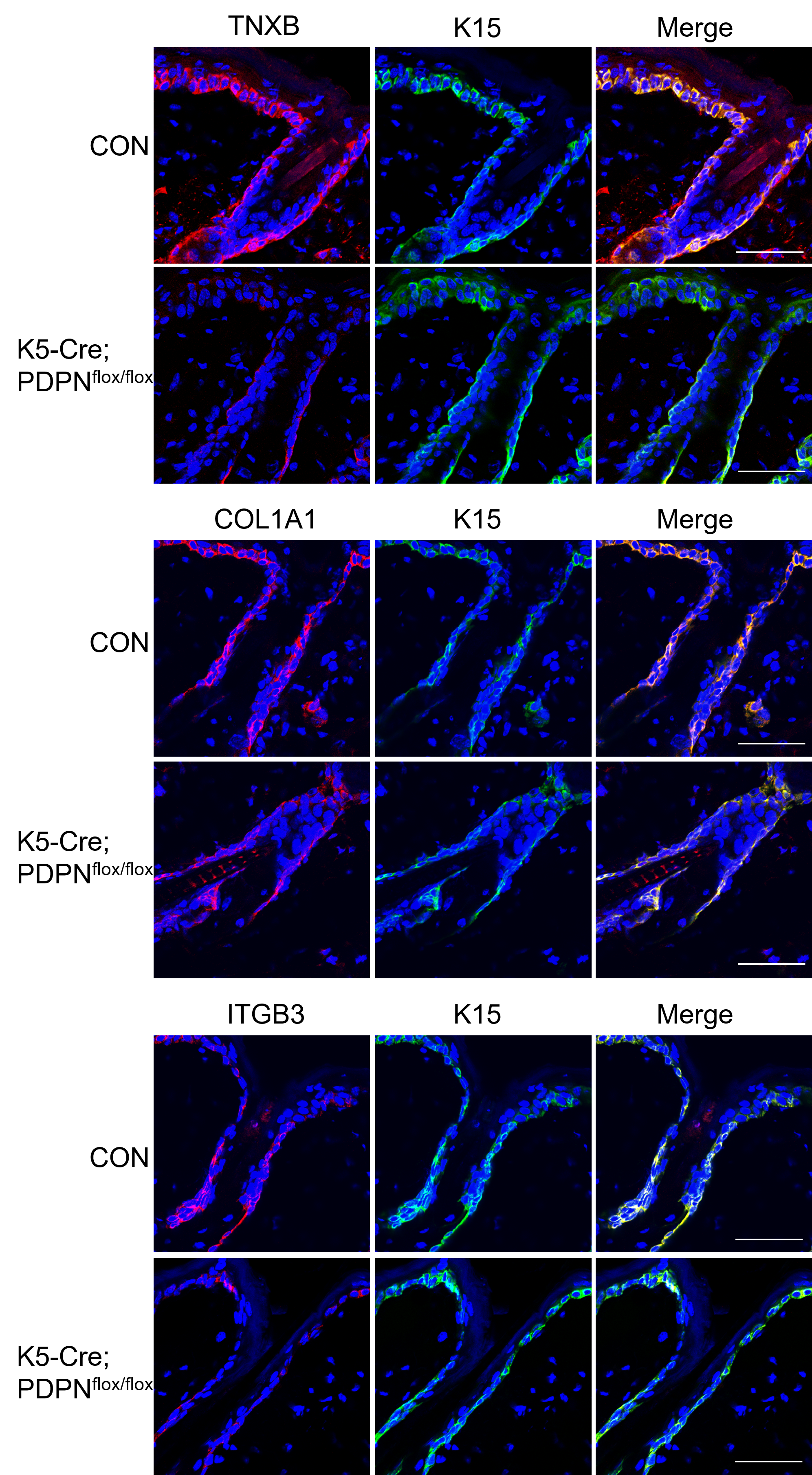

Supplement: S1 Fig — (TIF) [file pone.0219938.s001.tif]

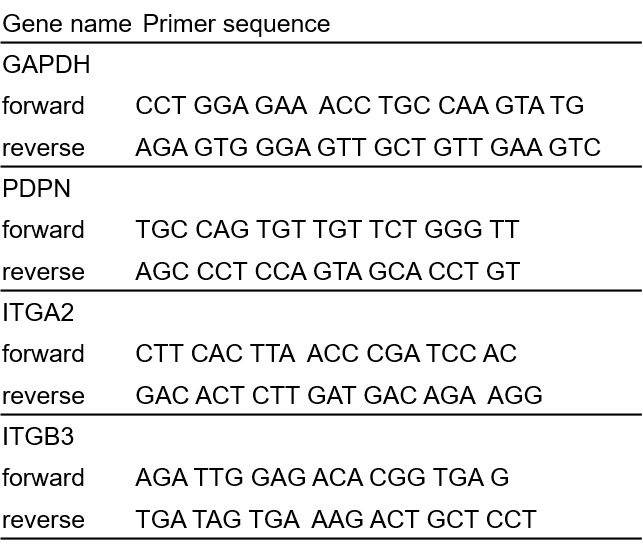

Supplement: S1 Table — (TIF) [file pone.0219938.s002.tif]
